# Supplementary material for: Heat adaptation of phage T7 under an extended genetic code
Source: Virus Evol. 2021 Dec 1;7(2):veab100. doi: 10.1093/ve/veab100 (PMC8923235; doi:10.1093/ve/veab100)
Supplement: veab100_Supp [file veab100_supp.zip › Sequencing_Analysis.zip!/Sequencing_Analysis/A1/evidence/JC_SIDE_2_54.html]

BRESEQ :: Evidence


| New junction evidence | | | | | | | | | | | |
| --- | --- | --- | --- | --- | --- | --- | --- | --- | --- | --- | --- |
|  | | seq id | position | reads (cov) | reads (cov) | score | skew | freq | annotation | gene | product |
| \* | ? | NC\_001604.1 | = 10002 | 2433 (0.790) | 15 (0.000) | 14/594 | 38.4 | 0.51% | intergenic (–/–) | *–/–* | –/– |
|  | ? | NC\_001604.1 | 10186 = | 3422 (1.120) |  |  |  |  | intergenic (–/–) | *–/–* | –/– |
| Rejected: Coverage evenness skew score above cutoff. | | | | | | | | | | | |
| Rejected: Frequency below/above cutoff threshold. | | | | | | | | | | | |

Only 100 of 3487 total aligned reads displayed.  

`AATCAAGGTGACTTCCTCTGGGTGTTGGGAGTGGCAGGGCGCTACGAACAATAAAGGGTACGGGCAGGTGTGGTGCAGCAATACCGGAAAGGTTGTCTACTGTCATCGCGTAATGTCTAATGCTCCGAAAGGTTCTACCGTCCTGCACTCCTGTGATAATCCATTATGTTGTAACCCTGAACACCTATCCATAGGAACTCCAAAAGAGAACTCCACTGACATGGTAAATAAGGGTCGCTCACACAAGGGGTATAAACTTTCAGACGAAGACGTAATGGCAATCATGGAGTCCAGCGAGTCCAATGTATCCTTAGCTCGCACCTATGG‑TGTCTCCCAACAGACTATTTGTGATATACGCAAAGGGAGGCGACATGGCAGGTTACGGCGCTAAAGGAATCCGAAAGGTTGGAGCGTTTCGCTCTGGCCTAGAGGACAAGGTTTCAAAGCAGTTGGAATCAAAAGGTATTAAATTCGAGTATGAAGAGTGGAAAGTGCCTTATGTAATTCCGGCGAGCAATCACACTTACACTCCAGACTTCTTACTTCCAAACGGTATATTCGTTGAGACAAAGGGTCTGTGGGAAAGCGATGATAGAAAGAA  >  NC_001604.1/9886‑10486`  

                                                                                                                                                                                                                                                                                                            `|`                                                                                                                                                                                                                                                                                                               
             `tCCTCTGGGTGTTGGGAGTGGCAGGGCGCTACGAAAAATAAAGGGTACGGGCAGGTGTGGTGCAGCAATACCGGAAAGGTTGTCTACTGTCATCGCGTAATGTCTAATGCTCCGAAAGGTTCTACCGTCCTGCACTCCTGTGATAATCCATTATGTTGTAACCCTGAACACCTATCCATAGGAACTCCAAAAGAGAACTCCACTGACATGGTAAATAAGGGTCGCTCACACAAGGGGTATAAACTTTCAGACGAAGACGTAATGGCAATCATGGAGTCCAGCGAGTCCAATGTATCCTTAg                                                                                                                                                                                                                                                                                                  <  1:427428/301‑1 (MQ=255)`  
                   `gggTGTTGGGAGTGGCAGGGCGCTACGAACAATAAAGGGTACGGGCAGGTGTGGTGCAGCAATACCGGAAAGGTTGTCTACTGTCATCGCGTAATGTCTAATGCTCCGAAAGGTTCTACCGTCCTGCACTCCTGTGATAATCCATTATGTTGTAACCCTGAACACCTATCCATAGGAACTCCAAAAGAGAACTCCACTGACATGGTAAATAAGGGTCGCTCACACAAGGGGTATAAACTTTCAGACGAAGACGTAATGGCAATCATGGAGTCCAGCGAGTCCAATGTATCCTTAGCTCGCa                                                                                                                                                                                                                                                                                            >  1:473663/1‑301 (MQ=255)`  
                     `gtgtacggAGTGGACGGGCGCTAAGAACAATAAAGGGTAAGTGCAGGGGTGGTGCAGCAATACCGGAAAGGTTGTCTACTGTCATCGCGTAATGTCTAATGCTCCGAAAGGTTCTACCGTCCTGCACTCCGGTGATAATCCATTATGTTGTAACCCTGAACACCTATCCATAGGAACTCCAAAAGAGAACTCCACTGACATGGTAAATAAGGGTCGCTCACACAAGGGGTATAAACTTTCAGACGAAGACGTCATGGCAATCATGGAGTCCAGCGAGTCCAATGTATCCTTAGCTCGCAcc                                                                                                                                                                                                                                                                                          <  1:347249/295‑1 (MQ=255)`  
                           `ggAGTGGCAGGGCGCTACGAACAATAAAGGGTACGGGCAGGTGTGGTGCAGCAATACCGGAAAGGTTGTCTACTGTCATCGCGTAATGTCTAATTCTCCGAAAGGTTCTACCGTCCTGCACTCCTGTGATAATCCATTATGTTGTAACCCTGAACACCTATCCATAGGAACTCCAAAAGAGAACTCCACTGACATGGTAAATAAGGGTCGCTCACACAATGGGTATAAACTTTCAGACGAAGACGTAATGGCAATCATGGAGTCCAGCGAGTCCAATGTATCCTTAGCTCGCACCTATGg‑t                                                                                                                                                                                                                                                                                   <  1:407187/301‑1 (MQ=255)`  
                            `gAGTGGCAGGGCGCTACGAACAATAAAGGGTACGGGCAGGTGTGGTGCAGCAATACCGGAAAGGTTGTCTACTGTCATCGCGTAATGTCTAATGCTCCGAAAGGTTCTACCGTCCTGCACTCCTGTGATAATCCATTATGTTGTAACCCTGAACACCTATCCATAGGAACTCCAAAAGAGAACTCCACTGACATGGTAAATAAGGGTCGCTCACACAAGGGGTATAAACTTTCAGACGAAGACGTAATGGCAATCATGGAGTCCAGCGAGTCCAATGTATCCTTAGCTCGCACCTATGg‑tg                                                                                                                                                                                                                                                                                  <  1:137913/301‑1 (MQ=255)`  
                            `gAGTGGCAGGGCGCTACGAACAATAAAGGGTACGGGCAGGTGTGGTGCAGCAATACCGGAAAGGTTGTCTACTGTCATCGCGTAATGTCTAATGCTCCGAAAGGTTCTACCGTCCTGCACTCCTGTGATAATCCATGATGTTGTAACCCTGAACACCTATCCATAGGAACTCCAAAAGAGAACTCCACTGACATGGTAAATAAGGGTCGCTCACACAAGGGGTATAAACTTTCAGACGAAGACGTAATGGCAATCATGGAGTCCAGCGAGTCCAATGTATCCTTAGCTCGCACCTATGg‑tg                                                                                                                                                                                                                                                                                  <  1:296294/301‑1 (MQ=255)`  
                                       `cgcTACGAACAATAAAGGGTACGGGCAGATGTGGTGCAGCAATACCGGAAAGGTTGTCTACTGTCATCGCGTAATTTCTAATGCTCCGAAAGGTTCTACCGTCCTGCACTCCTGTGATAATCCATTATGTTGTAACCCTGAACACCTATCCATAGGAACTCCAAAAGAGAACTCCACTGACATGGTAAATAAGGGTCGCTCACACAAGGGGTATAAACTTTCAGACGAAGACGTAATGGCAATCATGGAGTCCAGCGAGTCCAATGTATCCTTAGCTCGCACCTATGG‑TGTCTCCCAACAg                                                                                                                                                                                                                                                                       <  1:288606/301‑1 (MQ=255)`  
                                         `tgtataagagacaggggTACGGGCAGGTGTGGTGCAGCAATACCGGAAAGGTTGTCTACTGTCATCGCGTAATGTCTAATGCTCCGAAAGGTTCTACCGTCCTGCACTCCTGTGATAATCCATTATGTTGTAACCCTGAACACCTATCCATAGGAACTCCAAAAGAGAACTCCACTGACATGGTAAATAAGGGTCGCTCACACAAGGGGTATAAACTTTCAGACGAAGACGTAATGGCAATCATGGAGTCCAGCGAGTCCAATGTATCCTTAGCTCGCACCTATGG‑TGTCTCCCAACAGAc                                                                                                                                                                                                                                                                     <  1:360139/287‑1 (MQ=255)`  
                                         `cTACGAACAATAAACGGGACTGGCAGGTGTGGTGCAGCAATACAGGAAAGGTTGTCTACTGTCATCGCGTAATGTCTAATGCTACGAAAGGTTCTACCGGCCTGAACACCTGTGATAATCCATTATGTTGTAACCCTGAACACCTATCCATAGGAACTCCAAAAGAGAACTCCACTGACATGGTAAATAATGGTCGCTCACACAAGGGGTATAAACTTTCAGAAGAAGACGTAATGGCAATCATGGAATCCAGCGAGTCCAATGTATCCTTAGCTCGCAACTATGG‑TGTCTCCCAACAGAc                                                                                                                                                                                                                                                                     <  1:78666/301‑1 (MQ=255)`  
                                               `aaagagacaggTACGGGCAGGTGTGGTGCAGCAATACCGGAAAGGTTGTCTACTGTCATCGCGTAATGTCTAATGCTACGAAAGGTTCTACCGTCCTGCACTCCTGTGATAATCCATTATGTTGTAACCCTGAACACCTATCCATAGGAACTCCAAAAGAGAACTCCACTGACATGGTAAATAAGGGTCGCTCACACAAGGGGTATAAACTTTCAGACGAAGACGTAATGGCAATCATGGAGTCCAGCGAGTCCAATGTATCCTTAGCTCGCACCTATGG‑TGTCTCCCAACAGACTATTtg                                                                                                                                                                                                                                                               <  1:332359/292‑1 (MQ=255)`  
                                                  `aTAAAGGGTACGGGCAGGTGTGGTGCAGCAATACCGGAAAGGTTGTCTACTGTCATCGCGTAATGTCTAATGCTCCGAAAGGTTCTACCGTCCTGCACTCCTGTGATAATCCATTATGTTGTAACCCTGAACACCTATCCATAGGAACTCCAAAAGAGAACTCCACTGACATGGTAAATAAGGGTCGCTCACACAAGGGGTATAAACTTTCAGACGAAGACGTAATGGCAATCATGGAGTCCAGCGAGTCCAATGTATCCTTAGCTCGCACCTATGGCTGtctcttatacacatctccgag                                                                                                                                                                                                                                                             >  1:92020/1‑284 (MQ=255)`  
                                                    `aaaGGGTACGGGCAGGTGTGGTGCAGCAATACCGGAAAGGTTGTCTACTGTCATCGCGTAATGTCTAATGCTCCGAAAGGTTCTACCGTCCTGCACTCCTGTGATAATCCATTATGTTGTAACCCTGAACACCTATCCATAGGAACTCCAAAAGAGAACTCCACTGACATGGTAAATAAGGGTCGCTCACACAAGGGGTATAAACTTTCAGACGAAGACGTAATGGCAATCATGGAGTCCAGCGAGTCCAATGTATCCTTAGCTCGCACCTATGG‑TGTCTCCCAACAGACTATTTGTGata                                                                                                                                                                                                                                                          <  1:3904/301‑1 (MQ=255)`  
                                                        `ggTACGGGCAGGTGTGGTGCAGCAATACCGGAAAGGTTGTCTACTGTCATCGCGTAATGTCTAATGCTCCGAAAGGTTCTACCGTCCTGCACTCCTGTGATAATCCATTATGTTGTAACCCTGAACACCTATCCATAGGAACTCCAAAAGAGAACTCCACTGACATGGTAAATAAGGGTCGCTCACACAAGGGGTATAAACTTTCAGACGAAGACGTAATGGCAATCATGGAGTCCAGCGAGTCCAATGTATCCTTAGCTCGCACCTATGG‑TGTATCCCAACAGACtctgtctcttataca                                                                                                                                                                                                                                                      >  1:282/1‑287 (MQ=255)`  
                                                         `gTACGGGCAGGTGTGGTGCAGCAATACCGGAAAGGTTGTCTACTGTCATCGCGTAATGTCTAATGCTCCGAAAGGTTCTACCGTCCTGCACTCCTGTGATAATCCATTATGTTGTAACCCTGAACACCTATCCATAGGAACTCCAAAAGAGAACTCCACTGACATGGTAAATAAGGGTCGCTCACACAAGGGGTATAAACTTTCAGACGAAGACGTAATGGCAATCATGGAGTCCAGCGAGTCCAATGTATCCTTAGCTCGCACCTATGG‑TGTCTCCCAACAGACTATTTGTGATATACGc                                                                                                                                                                                                                                                     <  1:456188/301‑1 (MQ=255)`  
                                                            `cGGGCAGGTGTGGTGCAGCAATACCGGAAAGGTTGTCTACTGTCATCGCGTAATGTCTAATGCTCCGAAAGGTTCTACCGTCCTGCACTCCTGTGATAATCCATTATGTTGTAACCCTGAACACCTATCCATAGGAACTCCAAAAGAGAACTCCACTGACATGGTAAATAAGGGTCGCTCACACAAGGGGTATAAACTTTCAGACGAAGACGTAATGGCAATCATGGAGACCAGCGAGTCCAATGTATCCTTAGCTCGCACCTATGG‑TGTCTCCCCAAAGACTATTTGTGATATACGCaaa                                                                                                                                                                                                                                                  >  1:104059/1‑301 (MQ=255)`  
                                                             `gggCAGGTGTGGTGCAGCAATACCGGAAAGGTTGTCTACTGTCATCGCGTAATGTCTAATGCTCCGAAAGGTTCTACCGTCCTGCACTCCTGTGATAATCCATTATGTTGTAACCCTGAACACCTATCCATAGGAACTCCAAAAGAGAACTCCACTGACATGGTAAATAAGGGTCGCTCACACAAGGGGTATAAACTTTCAGACGAAGACGTAATGGCAATCATGGAGTCCAGCGAGTCCAATGTATCCTTAGCTCGCACCTATGG‑TGTCTCCCAACAGACTATTTGTGATATACGCAAAg                                                                                                                                                                                                                                                 >  1:112710/1‑301 (MQ=255)`  
                                                                        `gtgCAGCAATACCGGAAAGGTTGTCTACTGTCATCGCGTAATGTCTAATGCTCCGAAAGGTTCTACCGTCCTGCACTCCTGTGATAATCCATTATGTTGTAACCCTGAACACCTATCCATAGGAACTCCAAAAGAGAACTCCACTGACATGGTAAATAAGGGTCGCTCACACAAGGGGTATAAACTTTCAGACGAAGACGTAATGGCAATCATGGAGTCCAGCGAGTCCAATGTATCCTTAGCTCGCACCTATGG‑TGTCTCCCAACAGACTATTTGTGATATACGCAAAGGGAGGCGACAt                                                                                                                                                                                                                                      >  1:168976/1‑301 (MQ=255)`  
                                                                                  `aCCGGAAAGGTTGTCTACTGTCATCGCGTAATGTCTAATGCTCCGAAAGGTTCTACCGTCCTGCACTCCTGTGATAATCCATTATGTTGTAACCCTGAACACCTATCCATAGGAACTCCAAAAGAGAACTCCACTGACATGGTAAATAAGGGTCGCTCACACAAGGGGTATAAACTTTCAGACGAAGACGTAATGGCAATCATGGAGTCCAGCGAGTCCAATGTATCCTTAGCTCGCACCTATGG‑TGTCTCCCAACAGACTATTTGTGATATACGCAAAGGGAGGCGACATGGCAGGTTAc                                                                                                                                                                                                                            <  1:394769/301‑1 (MQ=255)`  
                                                                                           `gTTGTCTACTGTCATCGCGTAATGTCTAATGCTCCGAAAGGTTCTACCGTCCTGCACTCCTGTGATAATCCATTATGTTGTAACCCTGAACACCTATCCATAGGAACTCCAAAAGAGAACTCCACTGACATGGTCAATCAGGGTCGCTCACACAAGGGGTATAAACTTTCAGACGAAGACGTAATGGCAATCATGGAGTACAGCGAGTCCAATGTATCCTTAACTCGCACCTATGG‑TGTCTCCCAACAGACTATTTGTGATCTACGCAAAGGGAGGCGACATGGCAGGTTACGGCGCTaaa                                                                                                                                                                                                                   >  1:122745/1‑301 (MQ=255)`  
                                                                                                   `cTGTCATCGCGTAATGTCTAATGCTCCGAAAGGTTCTACCGTCCTGCACTCCTGTGATAATCCATTATGTTGTAACCCTGAACACCTATCCATAGGAACTCCAAAAGAGAACTCCCCTGACATGGTAAATAAGGGTCGCTCACACAAGCGGTATAAACTTTCAGACGAAGACGTAATGGCAATCATGGAGTCCAGCGAGTCCAATGTATCCTTAGCTAGCACCTATGG‑TGTCTCCCAACAGACTATTTGTGATATACGCAAAGGGAGGCGACATGGCAGGTTACGGCGCTAAAGGAATCCg                                                                                                                                                                                                           >  1:267767/1‑301 (MQ=255)`  
                                                                                                                `aTGTCTAATGCTCCGAAAGGTTCTACCGTCCTGCACTCCTGTGATAATCCATTATGTTGTAACCCTTAACACCTATCCATAGGAACTCCAAAAGAGAACTCCACTGACATGGTAAATAAGGGTCGCTCACACACGGGGTATAAACTTTCAGACGAAGACGTAATGGCAATCATGGCGTCCAGCGAGTCCCATGTATCCTTAGCTCGCACCTATGG‑TGTCTCCCAACAGACTATTTGTGATATACGCACAGGGAGGCGACATGGCAGTTTACGGCCCTAAAGGAATCCGAAAGGTTgcaccg                                                                                                                                                                                              >  1:59134/1‑296 (MQ=255)`  
                                                                                                                `aTGTCTAATGCTCCGAAAGGTTCTACCGTCCTGCACTCCTGTGATAATCCATTATGTTGTAACCCTGAACACCTATCCATAGGAACTCCAAAAGAGAACTCCACTGACATGGTAAATAAGGGTCGCTCACACAAGGGGTATAAACTTTCAGACGAAGACGTAATGGCAATCATGGAGTCCAGCGAGTCCAATGTATCCTTAGCTCGCACCTATGG‑TGTCTCCAAACAGACTATTTGTGATATACGCAAAGGGAGGCGACATGGCAGGTTACGGCGCTAACGGAATCCGAAAGGTTGGAGCg                                                                                                                                                                                              >  1:118521/1‑301 (MQ=255)`  
                                                                                                                    `cTAATGCTCCGAAAGGTTCTACCGTCCTGCACTCCTGTGATAATCCATTATGTTGTAACCCTGAACACCTATCCATAGGAACTACAAAAGAGAACTCCACTGACATGGTAAATAAGGGTCGCTCACACAAGGGGTATAAACTTTAAGACGAAGACGTAATGGCAATCATGGAGTCCAGCGAGTCCAATGTATACTTAGCTCGAACCTATGG‑TGTCTCCCAACAGACTATTTGTGATATACGCAAAGGGAGGCGACATGGCAGGTTACGGCGCTAAAGGAATCCGAAAGGTTGGAGCGTTTc                                                                                                                                                                                          >  1:128508/1‑301 (MQ=255)`  
                                                                                                                       `aTGGTCCGAAAGGTTCGTACGGCCGGGACTTCTGGGAGAAGCCATTAGGTGGTAACCATGAACACCTATCCATAGGAAATCCAGAAGAGAACTTCACTGACATGGTAAATAAGGGTCGCTAACACAAGGTGTATAAACTTTCAGACGAAGACGTAATGGCAATCATGGAGTCCAGCGAGTCCAATGTATCCTTAGCTCGCACCTATGG‑TGTCTCCCAACAGACTATTTGTGATATACGCAAAGGGAGGCGACATGGCAGGTTACGGCGCTAAAGGAATCCGAAAGGTTGGAGCGTTTCGct                                                                                                                                                                                       <  1:451401/301‑1 (MQ=255)`  
                                                                                                                       `aTGCTCCGAAAGGTTCTACCGTCCTGCACTCCTGTGATAATCCATTATGTTGTAACCCTGAACACCTATCCATAGGAACTCCAAAAGAGAACTCCACTGACATGGTAAATAAGGGTCGCTCACACAAGGGGTATAAACTTTCAGACGAAGACGTAATGGCAATCATGGAGTCCAGCGAGTCCAATGTATCCTTAGCTCGCACCTATGG‑TGTCTCCCAACAGACTATTTGTGCTATACGCAAAGGGAGGCGACATGGCAGGTTACGGCGCTAACGGAATCCGAAAGGTTGGAGCGTTTCGcc                                                                                                                                                                                       >  1:17900/1‑300 (MQ=255)`  
                                                                                                                          `cTCCGAAAGGTTCTACCGTCCTGCACTCCTGTGATAATCCATTATGTTGTAACCCTGAACACCTATCCATAGGAACTCCAAAAGAGAACTCCACTGACATGGTAAATAATGGTCGCTCACACAAGGGGTATAAACTTTCAGACGAAGACGTAATGGCAATCATGGAGTCCAGCGAGTCCAATGTATCCTTAGCTCGCACCTATGG‑TGTCTCCCAACAGACTATTTGTGATATACGCAAAGGGAGGCGACATGGCAGGTTACGGCGCTACAGGAATCCGAAAGGTTGGAGCgctgtctcttt                                                                                                                                                                                    >  1:366008/1‑291 (MQ=255)`  
                                                                                                                              `gAAAGGTTCTACCGTCCTGCACTCCTGTGATAATCCATTATGTTGTAACCCTGAACACCTATCCATAGGAACTCCAAAAGAGAACTCCACTGACATGGTAAATAAGGGTCGCTCACACAAGTGGTATAAACTTTCAGACGAAGACGTAATGGCAATCATGGAGTCCAGCTAGTCCAATGTATCCTTAGCTCGCACCTATGG‑TGTCTCCCAACAGACTATTTGTGATATACGCAAAGGGAGGCGACATGGCAGGTTACGGCGCTAAAGGAATCCGAAAGGTTGGATCGTTTCGCTCTGGCCt                                                                                                                                                                                >  1:435747/1‑301 (MQ=255)`  
                                                                                                                                  `ggTTCTACCGTCCTGCACTCCTGTGATAATCCATTATGTTGTAACCCTGAACACCTATCCATAGGAACTCCAAAAGAGAACTCCACTGACATGGTAAATAAGGGTCGCTCACACAAGGGGTATAAACTTTCAGACGAATACGTAATGGCAATCATGGAGTCCAGCGAGTCCAATGTATCCTTAGCTCGCACCTATGG‑TGTCTCCCAACAGACTATTTGTGATATACGCAAAGGGAGGCGACATGGCAGGTTACGGCGCTAAAGGAATCCGAAAGGTTGTAGCGTTTCGCTCTGGCCTagag                                                                                                                                                                            >  1:351489/1‑301 (MQ=255)`  
                                                                                                                                  `ggTTCTACCGTCCTGCACTCCTGTGATAATCCATTATGTTGTAACCCTGAACACCTATCCATAGGAACTCAAAAAGAGAACTCCACTGACATGGTAAATAAGGGTCGCTCACACAAGGGGTATAAACTTTCAGACGAAGACGTAATGGCAATCATGGAGTCCAGCGAGTCCAATGTATCCTTAGCTCGCACCTATGG‑TGTCTCCCAACAGACTATTTGTGATATACGCAAAGGGAGGCGACATGGCAGGTTACGGCGCTAAAGGAATCCGAAAGGTTGGAGCGTTTCGCTCTGGCCTagag                                                                                                                                                                            >  1:7385/1‑301 (MQ=255)`  
                                                                                                                                  `ggTTCTACCGTCCTGCACTCCTGTGATAATCCATTATGTTGTAAACCTGAACACCTATCCATAGGAACTCCAAAAGAGAACTCCACTGACATGGTAAATAAGGGTCGCTCACACAAGGGGTATAAACTTTCAGACGAAGACGTAATGGCAATCATGGAGTCCAGCGAGTCCAATGTATCCTTAGCTCGCACCTATGG‑TGTCTCCCAACAGACTATTTGTGATATACGCAAAGGGAGGCGACATGGCAGGTTACGGCGCTAAAGGAATCCGAAAGGTTGGAGCGTTTCGCTCTGGCCTagag                                                                                                                                                                            <  1:155807/301‑1 (MQ=255)`  
                                                                                                                                     `tCTACCGTCCTGCACTCCTGTGATAATCCATTATGTTGTAACCCTGAACACCTATCCATAGGAACTCCAAAAGAGAACTCCACTGACATGGTAAATAAGGGTCGCTCACACAAGGGGTATAAACTTTCAGACGAAGACGTAATGGCAATCATGGAGTCCAGCGAGTCCAATGTATCCTTAGCTCGCACCTATGG‑TGTCTCCCAACAGACTATTTGTGATATACGCAAAGGGAGGCGACATGGCAGGTTACGGCGCTAAAGGAATCCGAAAGGTTGGAGCGTTTCGCTATGGCCTAGAGGAc                                                                                                                                                                         <  1:53623/301‑1 (MQ=255)`  
                                                                                                                                          `gtataagagacagtgtgAAAATACATTATGTTGTAAACCAGAACACCAATCAAAAGGAACTCCAAAAGAGAACTCAACTGACATGGTAAATAAGGGTCGCTCACACAAGGGGTATAAACTTTCAGACGAAGACGTAATGGCAATCATGGAGCCCAGCGAGTCAAATGTATCCTAAGCGCGAACCTATGG‑TGTCTCCAAACAGACTATTTGTGATAGACGCAAAGGGAGGCGACATGGCGGGTTACGGCGATAAAGGAATCCGAAAGGTTGGAGTGTTTCGCTCTGGCCTAGAGGATAAGGt                                                                                                                                                                    <  1:235336/288‑1 (MQ=255)`  
                                                                                                                                                     `ccTGTGATAATCCATTATGTTGTAACCCTGAACACCTATCCATAGGAACTCCAAAAGAGAACTACACTGACATGGTAAATAAGGGTCGCTCACACAAGGGGTATAAACTTTCAGACGAAGACGTAATGGCAATCATGGAGTCAAGCGAGTCCAATGTATCCTTAGCTCGCACCTATGG‑TGTCTCCCAACAGACTATTTGTGATATACGCAAAGGGAGGCGACATGGCAGGTTACGGCGCTAAAAGAATCCTAAAGGTTGGAGCGTTTCGCTCTGGCCTAGAGGACAAGGGTTCACAGAAGt                                                                                                                                                         >  1:302303/1‑301 (MQ=255)`  
                                                                                                                                                           `aTAATCCATTATGTTGTAACCCTGAACACCTATCCATAGGAACTCCAAAAGAGAACTCCACTGACATGGTAAATAAGGGTCGCGCACACAAGGGGTATAAACTTTCAGACGAAGACGTAATGGCAATCATGGAGTCCAGCGAGTCCAATGTATCCTTAGCTCGCACCTATGG‑TGTCTCCCAACAGACTATTTGTGATATACGCAAAGGGAGGCGACATGGCAGGTTACGGCGCTAAAGGAATCCGAAAGGTTGGAGCGTTTCGCTCTGGCCTAGAGGACAAGGTTCCAAAGCAGTTGGAAt                                                                                                                                                   <  1:322505/301‑1 (MQ=255)`  
                                                                                                                                                           `aTAATCCATTATGTTGTAACCCTGAACACCTATCCATAGAAACTCCAAAAGAGAACTCCACTGACATGGTAAATAAGGGTCGCTCACACAAGGGGTATAAACTTTCAGACGAAGACGTAATGGCAATCATGGAGTCCAGCGAGTCCAATGTATCCTTAGCTCGCACCTATGG‑TGTCTCCCAACAGACTATTTGTGATATACGCAAAGGGAGGCGACATGGCAGGTTACGGCGCTAAAGGAATCCGAAAGGTTGGAGCGTTTCGCTCTGGCCTAGAGGACAAGGTTTCAAAGCAGTTGGAAt                                                                                                                                                   <  1:432633/301‑1 (MQ=255)`  
                                                                                                                                                               `tCCATTATGTTGTAACCCTGAACACCTATCCATAGGAACTCCAAAAGAGAACTCCACTGACATGGTAAATAAGGGTCGCTCACACAAGGGGTATAAACTTTCAGACGAAGACGTAATGGCAATCATGGAGTCCAGCGGGTCCAATGTATCCTTAGCTCGCACCTATGG‑TGTCTCCCAACAGACTATTTGTGATATACGCAAAGGGAGGCGACATGGCAGGTTACTGCGCTAAAGGAATCCGAAAGGTTGGAGCGTTTCGCTCTTGCCTAGAGGACAAGGTTTCAAAGCAGTTGGAATCaaa                                                                                                                                               >  1:230826/1‑301 (MQ=255)`  
                                                                                                                                                               `tCCATTATGTTGTAACCCTGAACACCTATCCATAGGAACTCCAAAAGAGAACTCCACTGACATGGTAAATAAGGGTCGCTCACACAAGGGGTATAAACTTTCAGACGAAGACGTAATGGCAATCATGGAGTCCAGCGAGTCCAATGTATCCTTAGCTCGCACCTATGG‑TGTCTCCCAACAGACTATTTGTGATATACGCAAAGGGAGGCGACATGGCAGGTTACGGCGATAAAGGAATCCGAAAGGTTGGAGCGTTTCGCTCTGGCCTAGAGGACAAGGTTTCAAAGcctgtctcttatac                                                                                                                                               >  1:376090/1‑288 (MQ=255)`  
                                                                                                                                                                `ccATTATGTTGTAACCCTGAACACCTATCCATAGGAACTCCAAAAGAGAACTCCACTGACATGGTAAATAAGGGTCGCTCACACAAGGGGTATAAACTTTCAGACGAAGACGTAATGGCAATCATGGAGTCCAGCGAGTCCAATGTATCCTTAGCTCGCACCTATGG‑TGTCTCCCAACAGACTATTTGTGATATACGCAAAGGGAGGCGACATGGCAGGTTACGGCGCTAAAGGAATCCGAAAGGTTGGAGCGTTTCGCTCTGGCCTAGAGGACAAGGTTTCAAAGCAGTTGGAATCaaaa                                                                                                                                              >  1:147336/1‑301 (MQ=255)`  
                                                                                                                                                                `ccATTATGTTGTAACCCTGAACACCTATCCATAGGAACTCCAAAAGAGAACTCCACTGACATGGTAAATAAGGGTCGCTCACACAAGGGGTATAAACTTTCAGACGAAGACGTAATGGCAATCATGGAGTCCAGCGAGTCCAATGTATCCTTAGCTCGCACCTATGG‑TGTCTCCCAACAGACTATTTGTGATATACGCAAAGGGAGGCGACATGGCAGGTTACGGAGCTAAAGGAATCCGAAAGGTTGGAGCGTTTCGCTCTGGCCTAGAGGACAAGGGTTCAAAGCAGTTGGAATCaaaa                                                                                                                                              >  1:495960/1‑301 (MQ=255)`  
                                                                                                                                                                  `cttATGTTGTAACCCTGAACACCTATCCATAGGAACTCCAAAAGAGAACTCCACTGACATGGTAAATAAGGGTCGCTCACACAAGGGTTATAAACTTTCAGACGAAGACGTAATGGCAATAATGGAGTCCAGCGAGTCCAATGTATCCTTAGCTCGCACCTATGG‑TGTCTCCCAACAGACTATTTGTGATATACTCAAAGGGAGGCGACATGGCAGGTTACGGCTCTAAAGGAATCCGAAAGGTTGGAGCGTTTCTCTCTGGCCTAGAGGACAAGTTTTCAAAGCAGTTGGAATCAAAAgg                                                                                                                                            <  1:193423/300‑1 (MQ=255)`  
                                                                                                                                                                  `aTTATGTTGTAACCCTGAACACCTATCCATAGGAACTCCAAAAGAGAACTCCACTGACATGGTAAATAAGGGTCGCTCACACAAGGGGTATAAACTTTCAGACGAAGACGTAATGGCAATCATGGAGTCCAGCGAGTCCAATGTATCCTTAGCTCGCACCTATGG‑TGTCTCCCAACAGACTATTTGTGATATACGCAAAGGGAGGCGACATGGCAGGTTACGGCGCTAAAGGAATCCGAAAGGTTGGAGCGTTTCGCTCTGGCCTAGAGGACAAGGTTTCAAAGCAGTTGGAATCAAAAgg                                                                                                                                            >  1:223408/1‑301 (MQ=255)`  
                                                                                                                                                                     `aTGTTGTAACCCTGAACACCTATCCATAGGAACTCCAAAAGAGAACTCCACTGACATGGTAAATAAGGGTCGCTCACACAAGGGGTATAAACTTTCAGACGAAGACGTAATGGCAATCATGGAGTCCAGCGAGTCCAATGTATCCTTAGCTCGCACCTATGG‑TGTATCCCAACAGACTATTTGTGATATACGCAAAGGGAGGCGACATGGCAGGTTACGGCGCTAAAGGAATCCGACAGGTTGGAGCGTTTCGCACTGGCCTAGAGGAAAAAGTTTAAAAGCAGTTTGAATCAAAAGGTAt                                                                                                                                         >  1:64191/1‑301 (MQ=255)`  
                                                                                                                                                                         `tgtAACCCTGAACACCTATCCATAGGAACTCCAAAAGAGAACTCCACTGACATGGTAAATAAGGGTCGCTCACACAAGGGGTATAAACTTTCAGACGAAGACGTAATGGCAATCATGGAGTCCAGCGAGTCCAATGTATACTTAGCTCGAACCTATGG‑TGTCTCCCAACAGACTATTTGTGATATACGCAAAGGGAGGCGACATGGCAGGTTACGGCGCCAAAGGAATCCGAAAGGTTGGAGCGTTTCGCTCTGGCCTAGAGGACAAGGTTTCAAAGCAGTTGGAATCAAAAGGTATTaaa                                                                                                                                     <  1:460304/301‑1 (MQ=255)`  
                                                                                                                                                                          `gtAACCCTGAACACCTATCCATAGGAACTCCAAAAGAGAACTCCACTGACATGGTAAATAAGGGTCGCTCACACAAGGGGTATAAACTTTCAGACGAAGACGTAATGGCAATCATGGAGTCCAGCGAGTCCAATGTATCCTTAGCTCGCACCTATGG‑TGTCTCCCAACAGACTATTTGTGATATACGCAAAGGGAGGCGACATGGCAGGTTACGGCGCTAAAGGAATCCGAAAGGTTGGAGCGTTTCGCTCTGGCCTAGAGGACAAGGTTTCAAAGcctgtctcttatacacatctccgag                                                                                                                                    >  1:215732/1‑277 (MQ=255)`  
                                                                                                                                                                          `gtAACACTGAACACCTATCCATAGGAACTCCAAAAGAGAACGCCACTGACATGGTAAATAAGGGTCGCTCACACAAGGGGTATAAACTTTCAGACGAAGACGTAATGGCAATCATGGAGTCCAGCGAGTCCAAGGTATCCTTAGCTCGCACCTATGG‑TGTCTCCCAACAGACTATTTGTGATATACGCAAAGGGAGGCGACATGGCAGGTTACGGCGCTAAAGGAATCCGAAAGGTTGGAGCGTTTCGCTCTGGCCTAGAGGACAAGGTTTCAAAGCAGTTGGAATCAAAAGGTATTAAAt                                                                                                                                    <  1:337430/301‑1 (MQ=255)`  
                                                                                                                                                                             `aCCCTGAACACCTATCCATAGGAACTCCAAAAGAGAACTCCACTGACATGGTCAATAAGGGTCGCTCACACAAGGGGTATAAACTTTCAGACGAAGACGTAATGGCAATCATGGAGTCCAGCGAGTCCAATGTATCCTTAGCTCGCACCTATGG‑TGTCTCCCAACAGACTATTTGTGATATACGCAAAGGGAGGCGACATGGCAGGTTACGGCGCTAAAGGAATCCGAAAGGTTGGAGCGTTTCGCTCTGGCCTAGAGGACAAGGTTTCAAAGCAGTTGGAATCAAAAGGTATTAAATTCg                                                                                                                                 >  1:326986/1‑301 (MQ=255)`  
                                                                                                                                                                             `aCCCTGAACACCTATCCATAGGAACTCCAAAAGAGAACTCCACTGACATGGTAAATAAGGGTCGCTCACACAAGGGGTATAAACTTTCAGACGAAGACGTAATGGCAATCATGGAGTCCAGCGAGTCCAATGTATCCTTAGCTCGCACCTATGG‑TGTCTCCCAACAGACTATTTGTGATATACGCAAAGGGAGGCGACATGGCAGGTTACGGCGCTAAAGGAATCCGAAAGGTTGGAGCGTTTCGCTCTGGCCTAGAGGACAAGGTTTCAAAGCAGTTGGAATCAAAAGGTATTAAATTCg                                                                                                                                 >  1:82463/1‑301 (MQ=255)`  
                                                                                                                                                                                 `agacacccTATCCATAGAAACTCCAAAAGAGAACTCCACTGACATGGTAAATAAGGGTAGCTCACACAAGGGGTATAAACTTTCAGACGAAGACGTAATGGCAATCATGGAGTCCAGCGAGTCCAATGTAGCCTTAGCTCGCACCTATGG‑TGTCTCCCAACAGACTATTTGTGATATACGCAAAGGGAGGCGACATGGCAGGTTACGGCGCTAAAGGAATCCGAAAGGTTGGAGCGTTTCGCTCTGGCCTAGAGGACAAGGTTTCAAAGCAGTTGGAATAAAAAGGTATTAAATTCGAGTa                                                                                                                             <  1:173622/295‑1 (MQ=255)`  
                                                                                                                                                                                      `cccTATCCATAGGAACTCCAAAAGATAACTCCACTGACATGGTAAATAAGGGTCGCTCACACAAGGGGTATAAACTTTCAGACGAAGACGTAATGGCAATCATGGATTCCAGCGAGTCCAATGTATCCTTAGCTCGCACCTATGG‑TGTCTCCCAACAGACTATTTGTGATATACGCAAAGGGAGGCGACATGGCAGGTTACGGCGCTAAAGGAATCCGAAAGGTTGGAGCGTTTCGCTCTGGCCTAGAGGACAAGGTTTCAAAGCAGTTGGAATCAAAAGGTATTAAATTCGAGTATGAag                                                                                                                        <  1:342601/300‑1 (MQ=255)`  
                                                                                                                                                                                          `aTCCATAGGAACTCCAAAAGAGAACTCCACTGACATGGTAAATAAGGGTCGCTCACACAAGGTGTATAAACTTTCAGACGAAGACGTAATGGCAATCATGGAGTCCAGCGAGTCCAATGTATCCTTAGCTCGCACCTATGG‑TGTCTCCCAACAGACTATTTGTGATATACGCAAAGGGAGGCGACATGGCAGGTTACGGCGCTAAAGGAATCCGAAAGGTTGGAGCGTTTCGCTCTGGCCTAGAGGACAAGGTTTCAAAGCAGTTGGAATCAAAAGGTATTAAATTCGAGTATGAAGAGTg                                                                                                                    >  1:308204/1‑301 (MQ=255)`  
                                                                                                                                                                                          `aTCCATAGGAACTCCAAAAGAGAACTCCACTGACATGGTAAATAAGGGTCGCTCACACAAGGGGTATAAACTTTCAGACGAAGACGTAATGGCAATCATGGAGTCCAGCGAGTCCAATGTATCCTTAGCTCGCACCTATGG‑TGTCTCCCAACAGACTATTTGTGATATACGCAAATGGAGGCGACATGGCAGGTTACGGCGCTAAAGGAATCCGAAAGGTTGGAGCGTTTCGCTCTGGCCTAGAGGACAAGGTTTCAAAGCAGTTGGAATCAAAAGGTATTAAATTCGAgctgtctcttat                                                                                                                    >  1:100040/1‑290 (MQ=255)`  
                                                                                                                                                                                             `cATAGGAACTCCAAAAGAGAACTCCACTGACATGGTAAATAAGGGTCGCTCACACAAGGGGTATAAACTTTCAGACGAAGACGTAATGGCAATCATGGAGTCCAGCGAGTCCAATGTATCCTTAGCTCGCACCTATGG‑TGTCTCCCAACAGACTATTTGTGATATACGCAAAGGGAGGCGACATGGCAGGTTACGGCGCTAAAGGAATCCGAAAGGTTGGAGCGTATCGCTCTGGCCTAGAGGACAAGGTTTCAAAGCAGTTGGAATCAAAAGGTATTAAATTCGAGTATGAAGAGTGGaa                                                                                                                 <  1:132774/301‑1 (MQ=255)`  
                                                                                                                                                                                             `aaTAGGAACTCCAAAAGAGAACTCCAATTATATGGTAAATAAGGGTCGCGCACACAAGGGGTATAAACTTTCAGACGAAGACGTAATTGCAATCATGGAGTCCAGCGAGTGCAATGTATCCTTAGCTCGCACCTATGG‑TTTCTCCCAACAGACTATTGGTGATATACGCAAAGGGAGGCGACATGGCAGGTTACTGCGCTAAAGGAATCCGAAAGGTTGGAGCGTTTCGCTCTGGCCTAGAGGACAAGGTTTCAAAGCAGTTGGAATCAAAAGGTATTAAATTCGAGTATGAAGAGTGGaa                                                                                                                 <  1:202006/300‑1 (MQ=255)`  
                                                                                                                                                                                               `tAGGAACTCCAAAAGAGAACTCCACTGACATGGTAAATAAGGGTCGCTCACACAAGGGGTATAAACTTTCAGACGAAGACGTAATGGCAATCATGGAGTCCAGCTAGTCCAATGTATCCTTAGCTCGCACCTATGG‑TGTCTCCCAACAGACTATTTGTGATATACGCAAAGGGAGGCGACATGGCAGGTTACGGCGCTAAAGGAATCCGAAAGGTTGGAGCGTTTCGCTCTGGCCTAGAGGACAAGGTTTCAAAGCAGTTGGAATCAAAAGGTATTAAATTCGAGTATGACGAGTGGAAAg                                                                                                               >  1:316803/1‑301 (MQ=255)`  
                                                                                                                                                                                                     `cTCCAAAAGAGAACTCCACTGACATGGTAAATAAGGGTCGCTCACACAAGGGGTATAAACTTTCAGACGAAGACGTAATGGCAATTATGGAGTCCAGCGAGTCCAATGTATCCTTAGCTCGCACCTATGG‑TGTCTCCCAACAGACTATTTGTGATATACGAAAAGGGAGGCGACATGGCAGGTTACGGCGCTAAAGGAATCCGAAAGGTTGGAGCGTTTCGCTCTGGCCTAGAGGACAAGGTTTCAAAGCAGTTGGAATCAAAAGGTATTAAATTCGAGTATGAAGAGTGGAAAGTGCCtt                                                                                                         >  1:398463/1‑301 (MQ=255)`  
                                                                                                                                                                                                        `cAAAAGAGAACTCCACTGACATGGTAAATAAGGGTCGCTCACACAAGGGGTATAAACTTTCAGACGAAGACGTAATGGCAATCATGGAGTCCAGCGAGTCCAATGTATCCTTAGCTCGCACCTATGG‑TGTCTCCCAACAGACTATTTGTGATATACGCAAAGGGAGGCGACATGGCAGGTTACGGCGCTAAAGGAATCCGAAAGGTTGGAGCGTTTCGCTCTGGCCTAGAGGACAAGGTTTCAAAGCAGTTGGAATCAAAAGGTATTAAATTCGAGTATGAAGAGTGGAAAGTGCCTTATg                                                                                                      <  1:29223/301‑1 (MQ=255)`  
                                                                                                                                                                                                         `aaaaGAGAACCCCACTGACATGGTAAATAAGGGTCGCTCACACAAGGGGTATAAACTTTCAGACTAAGACGTAATGGCAACCATGGAGTCCAGCGAGTCCAATGTATCCTGAGCTCGCACCTATGG‑TGTCTCCCAACAGACTATTTGTGATATACGCAAAGGGAGGCGACATGGCAGGTTACGGCGCTAAAGGAATCCGAAAGGTTGGAGCGTTTCGCTCTGGCCTAGAGGACAAGGTTTCAAAGCAGTTGGAATCAAAAGGTATTAAATTCGAGTATGAAGAGTGGAAAGTGCCTTATGt                                                                                                     <  1:141714/301‑1 (MQ=255)`  
                                                                                                                                                                                                              `ggaACTCCACTGACATGGTAAATAAGGGTCGCTCACACAAGGGGTATAAACTTTCAGACGAAGACGTAATGGCAATCATGGAGTCCAGCGAGTCCAATGTATCCTTAGCTCGCACCTATGG‑TGTCTCCCAACAGACTATTTGTGATATACGCAAAGGGAGGCGACCTGGCAGGTTACGGCGCTAAAGGAATCCGAAAGGTTGGAGCGTCTCGCTCTGGCCTAGAGGACAAGGTTTCAAAGCAGTTGGAATCAAAAGGTATTAAATTCGAGTATGAAGAGTGGAAAGTGCCTTATGTAATTc                                                                                                >  1:87369/2‑301 (MQ=255)`  
                                                                                                                                                                                                              `agaatctcACTGACATGGTAAATAAGGGTTGCGCACACAATGGGTATAAACTTTCAGTCGAAGACGTAATGGCAATCATGGAGTCCAGCGAGTCCAATGTATCCTTAGCTCGCACCTATGG‑TGTCTCCCAACAGTCTATTTGTGATATACGCAAAGGGAGGCGACATGGCAGGTTACGGCGCTAAAGGAATCCTAAAGGTTGGAGCGTTTCGCTCTGGCCTAGAGGACAAGGTTTCAAAGCAGTTGGAATCAAAAGGTATTAAATTCGAGTATGAAGAGTGGAAAGTGCCTTATGTAATTc                                                                                                <  1:386926/294‑1 (MQ=255)`  
                                                                                                                                                                                                              `agaACACCACTGTAATGTGTATTAAGGGTAGCTCACACAAGGTGAATAAACTTTCAGAAGAAGACGTAATCGCAATCATGTAGTCCAGCGAGACCAATGTATCCTTAGTTCGCACATATGG‑TGTCTCCCAACAGACTAGTTGTGATATACGCAAAGGGAGGCGACATGGCAGGTTACGGCTCTAAAGGAATCCGAAAGGTTGGAGTGTTTCGCTCTGGCCTAGAGGACAAGGTGTCAAAGCATTTGGAATCAAAAGGTATTAAATTCGAGTATGAAGAGTGGAAAGTGCCTTATGTAATTc                                                                                                <  1:249141/301‑1 (MQ=255)`  
                                                                                                                                                                                                                 `aCTCCACTGACATGGTAAATAAGGGTCGCTCACACAAGGGGTATAAACTTTCAGACGAAGACGTAATGGCAATCATGGAGTCCAGCGAGTCCAATGTATCCTTAGCTCGCACCTATGG‑TGTCTCCCAACAGACTATTTGTGATATACGCAAAGGGAGGCGACATGGCAGGTTACGGCGCTAAAGGAATCCGAAAGGTTGGAGCGTTTCGCTCTGGCCTAGAGGACAAGGTTTCAAAGCAGTTGGAATCAAAAGGTATTAAATTCGAGTATGAAGAGTGGAAAGTGCCTTATGTAAtctgtc                                                                                             >  1:413497/1‑296 (MQ=255)`  
                                                                                                                                                                                                                 `aCTCCAATGACATGGTAAATAAGGGTCGCTCACACAAGGGGTATAAACTTTCAGACGAAGACGTAATGGCAATCATGGAGTCCAGCGAGTCCAATGTATCCTTAGCTCGCAACTATGG‑TGTCTCCCAACAGACTATTTGTGATATACGCAAAGGGAGGCGACATGGCAGGTTACGGCGCTAAAGGAATCCGAAAGGTTGGAGCGTTTCGCTCTGGCCTAGAGGACAAGGTTTCAAAGCAGTTGGAATCAAAAGGTATTAAATTCGAGTATGAAGAGTGGAAAGTGCCTTATGTAATTCCgg                                                                                             <  1:188365/301‑1 (MQ=255)`  
                                                                                                                                                                                                                    `ccACTGACATGGTAAATAAGGGTCGCTCACACAAGGGGTATAAACTTTCAGACGAAGACGTAATGGCCATCATGGAGTCCAGCGAGTCCAATGTATCCTTAGCTCGCACCTATGG‑TGTCTCCCAACAGACTATTTGTGATATACGCAAAGGGAGGCGACATGGCAGGTTACGGCGCTCAAGGAATCCGAAAGGTTGGAGCGTTTCGCTCT‑GCCTAGAGGACAAGGTTTCAAAGCAGTTGGAATCCAACGGTATTAAATTCGAGTATGAAGAGTGGAAAGTGCATTATGTAATTCCGGCGAg                                                                                         >  1:67859/1‑301 (MQ=255)`  
                                                                                                                                                                                                                    `ccACTGACATGGTAAATAAGGGTCGCTCACACAAGGGGTATAAACTTTCAGACGAAGACGTAATGGCAATCATGGAGTCCAGCGAGTCCAATGTATCCTTAGCTCGCACCTATGG‑TGTCTCCCAACATACTATTTGTGATATACGCAAAGGGAGGCGACATGGCAGGTTACGGCGCTAAAGGAATCCGAAAGGTTGGAGCGTTTCGCTCTGGCCTAGAGGACAAGGTTTCAAAGCAGTTGGAATCAAAAGGTATTAAATTCGAGTATGAAGAGTGGAAAGTGCCTTCTGTAATTCCGGCGa                                                                                          >  1:245208/1‑301 (MQ=255)`  
                                                                                                                                                                                                                    `ccACTGACATGGTAAATAAGGGTCGCTCACACAAGGGGTATAAACTTTCAGACGAAGACGTAATGGCAATCATGGAGTCCAGCGAGTCCAATGTATCCTTAGCTCGCACCTATGG‑TGTCTCCCAACAGACTATTTGTGATATACGCAAAGGGAGGCGACATGGCAGGTTACGGCGCTAAAGGAATCCGAAAGGTTGGAGCGTTTCGCTCTGGCCTAGAGGACAAGGTTTCAAAGCAGTTGGAATCAAAAGGTATTAACTTCGAGTATGAagctgtctcttatacacatctccgatcccacc                                                                                          >  1:312555/1‑271 (MQ=255)`  
                                                                                                                                                                                                                    `ccACTGACATGGTAAATAAGGGTCGCTCACACAAGGGGTATAAACTTTCAGACGAAGACGTAATGGCAATCATGGAGTCCAGCGAGTCCAATGTATCCTTAGCTCGCACCTATGG‑TGTCTCCCAACAGACTATTTGTGATATACGCAAAGGGAGGCGACATGGCAGGTTACGGCGCTAAAGGAATCCGAAAGGTTGGAGCGTTTCGCTCTGGCCTAGAGGACAAGGGTTCAAAGCAGTTGGAATCAAAAGGTATTAAATTCGAGTATGAAGAGTGGAAAGTGCCTTATGTAATTCCGGCgg                                                                                          >  1:354832/1‑300 (MQ=255)`  
                                                                                                                                                                                                                           `cATGGTAAATAAGGGTCGCTCACACAAGGGGTATAAACTTTCAGACGAAGACGTAATGGCAATCATGGAGTCCAGCGAGTCCAATGTATCCTTAGCTCGCACCTATGG‑TGTCTCCCAACAGACTATTTGTGATATACGCAAAGGGAGGCGACATGGCAGGTTACGGCGCTAAAGGAATCCGAAAGGTTGGAGCGTTTCGCTCTGGCCTAGAGGACAAGGTTTCAAAGCAGTTGGAATCAAAAGGTATTAAATTCGAGTATGAAGAGTGGAAAGTGCCTTATGtctgtctcttatacacatt                                                                                   >  1:277384/1‑283 (MQ=255)`  
                                                                                                                                                                                                                              `ggTAAATAAGGGTCGCTCACACAAGGGGTATAAACTTTCAGACGAAGACGTAATGGCAATCATGGAGTCCAGCGAGTCCAATGTATCCTTAGCTCGCACCTATGG‑TGTCTCCCAACAGACTATTTGTGATATACGCAAAGGGAGGCGACATGGCAGGTTACGGCGCTAAAGGAATCCGAAAGGTTGGAGCGTTTCGCTCTGGCCTAGAGGACAAGGTTTCAAAGCAGATGGAATCAAAAGGTATTAAATTCGAGTATGAAGAGTGGAAAGTGCCTTATGTAATTCCGGCGAGCAATCacac                                                                                >  1:487001/1‑301 (MQ=255)`  
                                                                                                                                                                                                                              `aagaaaTAAGGGTCGCTCAAACAAGGGGTATAAACTTTCAGACGAAGACGTAATGGCAAGCATGGAGTCCAGCGAGTCCAATGGATCCTTAGCTCGCACCTATGG‑TGTCGCCCAACAGACTATTTGTGATATACGCAAAGGGAGGCGACATGGCAGGTTACGGCGCTAAAGGAATCCGAAAGGTTGGAGCGTTTCGATCTGGCCTAGAGGACAAGGTTTCAAAGCAGTTGGAATCAAAAGGTATTAAATTCGAGTATGAAGAGTGGAAAGTGCCTTATGTAATTCCGGCGAGCAATCacac                                                                                <  1:371080/298‑1 (MQ=255)`  
                                                                                                                                                                                                                               `gTAAATAAGGGTCGCTCACACAAGGGGTATAAACTTTCAGACGAAGACGTAATGGCAATCATGGAGTCCAGCGAGTCCAATGTATCCTTAGCTCGCACCTATGG‑TGTCTCCCAACAGACTATTTGTGATATACGCAAATGGAGGCGACATGGCAGGTTACGGCGCTAAAGGAATCCGAAAGGTTGGAGCGTTTCGCTCTGGCCTAGAGGACAAGATTTCAAAGCAGTTGGAATCAAAAGGTATTAAATTCGAGTATGAAGAGTGGAAAGTGCCTTATGTAATTCCGGCGAGCAATCACACt                                                                               >  1:206194/1‑301 (MQ=255)`  
                                                                                                                                                                                                                               `gTAAATAAGGGTCGCTCACACAAGGGGTATAAACTTTCAGACGAAGACGTAATGGCAATCATGGAGTCCAGCGAGTCCAATGTATCCTTAGCTCGCACCTATGG‑TGTCTCCCAACAGACTATTTGTGATATACGCAAAGGGAGGCGACATGGCAGGTTACGGCGCTAAAGGAATCCGAAAGGTTGGAGCGTTTCGCTCTGGCCTAGAGGACAAGGTTTCAAAGCAGTTGGAATCAAAAGGTATTAAATTCGAGTATGAAGAGTGGAAAGTGCCTTATGTAATTCCGGCGAGCAATCACACt                                                                               >  1:183866/1‑301 (MQ=255)`  
                                                                                                                                                                                                                               `gTAAATAAGGGTCGCTCACACAAGGGGTATAAACTTTCAGACGAAGACGTAATGGCAATCATGGAGTCCAGCGAGTCCAATGTATCCTTAGCTCGCACCTATGG‑TGTCTCCCAACAGACTATTTGTGATATACGCAAAGGGAGGCGACATGGCAGGTTACGGCGCTAAAGGAATCCGAAAGGTTGGAGCGTTTCGCTCTGGCCTAGAGGACAAGGTTTCAAAGCAGTTGGAATCAAAAGGTATTAAATTCGAGTATGAAGAGTGGAAAGTGCCTTATGTAATTCCGGCGAGCAATCACACt                                                                               >  1:422550/1‑301 (MQ=255)`  
                                                                                                                                                                                                                                 `aaaTAAGGGTCGCTCACACAAGGGGTATAAACTTTCAGACGAAGACGTAATGGCAATCATGGAGTCCAGCGAGTCCAATGTATCCTTAGCTCGCACCTATGG‑TGTCTCCCAACAGACTATTTGTGATATACGCAAAGGGAGGCGACATGGCAGGTTACGGCGCTAAAGGAATCCGAAAGGTTGGAGCGTTTCGCTCTGGCCTAGAGGACAAGGTTTCAAAGCAGTTGGAATCAAAAGGTATTAAATTCGAGTATGAAGGGTGGAAAGTGCCTTACGTAATTCAGGCGAGAAATTACACTTa                                                                             >  1:209828/1‑301 (MQ=255)`  
                                                                                                                                                                                                                                 `aaaTAAGGGTCGCTCACACAAGGGGTATAAACTTTCAGACGAAGACGTAATGGCAATCATGGAGTCCAGCGAGTCCAATGTATCCTTAGCTCGCACCTATGG‑TGTCTCACAACAGACTATTTGTGATATACGAAAAGGGAGGCGACATGGCAGGTTACGGCGCTAAAGGAATCCGAAAGGTTGGAGCGTTTCGCTCTGGCCTAGAGGACAAGGTTTCAAAGCAGTTGGAATCAAAAGGTATTAAATTCGAGTATGAAGAGTGGAAAGTGACTAAAGTAATTCCGGCGAGAAATTACACTTa                                                                             >  1:252710/1‑301 (MQ=255)`  
                                                                                                                                                                                                                                  `aagagacagagatCAAACAAGGGGTATAAACTTTCAGACTAAGACGTAATGGCAATCATGGAGTCCAGCGAGTCCAATGGATCCTTAGCTCGCACCTATGG‑TGTCACCCAACAGACTATTTGTGATATACGCAAAGGGAGGCGACATGGCAGGTTACGGCGATAAAGGAATCCGAAAGGTTGGAGCGTTTCGCTCTGGCCTAGAGGACAAGGTTTCAAAGCAGTTGGAATCAAAAGGTATTAAATTCGAGTATGAAGAGTGGAAAGTGCCTTATGTAATTCCGGCGAGCAATCACACTTac                                                                            <  1:465588/289‑1 (MQ=255)`  
                                                                                                                                                                                                                                    `aaaGGGTCGCTCACACAAGGGTTATAAACTTTCAGACGAAGACGTAATGGCAATCATGGAGTCCAGCGAGTCCAATGTATCATTAGCTCGCACCTATGG‑TGTCTCCCAACAGACTATTTGTGATATACGCAAAGGGAGGCGACATGGCAGGTTACGGCGCTAAAGGAATCCGAAAGGTTGGAGCGTTTCGCTCTGGCCTAGAGGACAAGGTTTCAAAGCAGTTGGAATCAAAAGGTATTAAATTCGAGTATGAAGAGTGGAAAGTGCCTTATGTAATTCCGGCGAGCAATCACACTTacac                                                                          <  1:197269/300‑1 (MQ=255)`  
                                                                                                                                                                                                                                    `aaaGGGTCGCTCACACAAGGGGTATAAACTTTCAGACGAAGACGTAATGGCAATCATGGAGTCCATCGAGTCCAATGTATCCTTAGCTCGCACCTATGG‑TGTCTCCCAACAGACTATTTGTGATATACGCAAAGGGAGGCGACATGGCAGGTTACGGCGCTAAAGGAATCCGAAAGGTTGGAGCGTTTCGCTCTGGCCTAGAGGACAAGGTTTCAAAGCAGTTGGAATCAAAAGGTATTAAATTCGAGTATGAAGAGTGGAAAGTGCCTTATGTAATTCCGGCGAGCAATCACACTTacac                                                                          <  1:478602/300‑1 (MQ=255)`  
                                                                                                                                                                                                                                    `aaaGGGTCGCTCACACAAGGGGTATAAACTTTCAGACGAAGACGTAATGGCAATCATGGAGTCCAGCGAGTCCAATGTATCCCTAGCTCGCACCTATGG‑TGTCTCCCAACAGACTATTTGTGATATACGCAAAGGGAGGCGACATGGCAGGTTACGGCGCTAAAGGAATCCGAAAGGTTGGAGCGTTTCGCTCTGGCCTAGAGGACAAGGTTTCAAAGCAGTTGGAATCAAAAGGTATTAAATTCGAGTATGAAGAGTGGAAAGTGCCTTATGTAATTCCGGCGAGCAATCACACTTacac                                                                          <  1:165484/300‑1 (MQ=255)`  
                                                                                                                                                                                                                                      `aGGGTCGCTCACACAAGGGGTATAAACTTTCAGACGAAGGCGTAATGGCAATCATGGAGTCCAGCGATTACAATGGATCCTGAGCGCGCACCTATGG‑TGTATCCCAACAGACTATTTGTGATATACGCAAAGGGAGGTGACATGGCAGGTTACGGCGCTAAAGGTATCCGAAAGGTTGGAGCGTTTCGCTCTGGCATAGAGGACAAGGTTTCAAAGCAGTTGGAATCAAAAGGTATTAAATTTGAGTATGAAGAGTGGAAAGTGCCTTATGTAATTCCGGCGAGCAATCACACTTACACTc                                                                        <  1:40325/301‑1 (MQ=255)`  
                                                                                                                                                                                                                                       `gggTCGCTCACACAAGGGGTATAAACTTTCAGACGAAGACGTAATGGCAATCATGGAGTCCAGAGAGTCCAATGTATCCTTAGCTCGCACCTATGG‑TGTCTCCCAACAGACTATTTGTGATATACGCAAAGGGAGGCGACATGGCAGGTTACGGCGCTAAAGGAATCCGAAAGGTTGGAGCGTTTCGCTCTGGCCTAGAGGACAAGGTTTCAAAGCAGTTGGAATCAAAAGGTATTAAATTCGAGTATGAAGAGTGGAAAGTGCCTTAAGTAATTACGGCGAGCAATAACACTTacacctg                                                                       >  1:219380/1‑298 (MQ=255)`  
                                                                                                                                                                                                                                            `gCTCACACAAGGGGTATAAACTTTCAGACGAAGACGTAATGGCAATCATGGAGTCCAGCGAGACCAATGTATCCTTAGCTCGCACCTATGG‑TGTCTCCCAACAGACTATTTGTGATATACGCAAAGGGAGGCGACATGGCAGGTTACGGCGCTAAAGGAATCCGAAAGGTTGGAGCGTTTCGCTCTGGCCTAGAGGACAAGGTTTCAAAGCAGTTGGAATCAAAAGGTATTAAATTCGAGTATGAAGAGTGGAAAGTGCCTTATGTAATTCCGGCGAGCAATCACACTTACACTCCAGAct                                                                  <  1:447566/301‑1 (MQ=255)`  
                                                                                                                                                                                                                                              `acacacaAGGGGTATAAACTTTCAGACGAAGACGTAATGGCAATCATGGAGTCCAGCGAGTCCAATGTAGCCTTAGCTCGCACCTATGG‑TGTCTCCCAACAGACGATTTGTGATATACGCAAAGGGAGGCGACATGGCAGGTTACGGCGCTAAAGGAATCCGAAAGGTTGGAGCGTTTCGCTCTGGCCTAGAGGACAAGGTTTCAAAGCAGTTGGAATCAAAAGGTATTAAATTCGAGTATGAAGAGTGGAAAGTGCCTTATGTAATTCCGGCGAGCAATCACACTTACACTCCAGActtc                                                                <  1:469566/300‑1 (MQ=255)`  
                                                                                                                                                                                                                                                 `cacaAGGGGTATAAACTTTCAGACGAAGACGTAATGGCAATCATGGAGTCCAGCGAGTCCAATGTATCCTTAGCTCGCACCTATGG‑TGTCTCCCAACAGACTATTTGTGATATACGCAAAGGGAGGCGACATGGCAGGTTACGGCTCTAAAGGAATCCGAAAGGTTGGAGCGTTTCGCTCTGGCCTAGAGGACAAGGTTTCAAAGCAGTTGGAATCACAAGGTATTAAATTCGAGTATGAAGAGTGGAAAGTGCCTTATGTAATTCCGGCGAGCAATCACACTTACACTCCAGACTTCTTa                                                             >  1:381999/1‑301 (MQ=255)`  
                                                                                                                                                                                                                                                       `gggTATAAACTTTCAGACGAAGACGTAATGGCAATCATGGAGTCCAGCGAGTCCAATGTATCCTTAGCTCGCACCTATGG‑TGTCTCCCAACAGACTATTTGTGATATACGCAAAGGGAGGCGACATGGCAGGTTACGGCGCTAAAGGAATCCGAAAGGTTGGAGCGTTTCGCTCTGGCCTAGAGGACAAGGTTTCAAAGCAGTTGGAATCAAAAGGTATTAAATTAGAGTATGAAGAGTGGAAAGTGCCTTATGTAATTCCGGCGAGCAATCACACTTACACTCCAGACTTCTTACTTCCa                                                       >  1:227130/1‑301 (MQ=255)`  
                                                                                                                                                                                                                                                        `ggTATAAACTTTCAGACGAAGACGTAATGGCAATCATGGAGTCCAGCGAGTCCAATGTATCCTTAGCTCGCACCTATGG‑TGTCTCCCAACAGACTATTTGTGATATACGCAAAGGGAGGCGACATGGCAGGTTACGGCGCTAAAGGAATCCGAAAGGTTGGAGCGTGTCGCTCTGGCCTAGAGGACAAGGTTTCAAAGCAGTTGGAATAAAAAGGTATTAAATTCGAGTATGAAGAGTGGAAAGTGCCTTATGTAATTACGGAGAGCAATCAAAAGTACACTCCAGACATTTTACTTCCaa                                                      >  1:95487/1‑301 (MQ=255)`  
                                                                                                                                                                                                                                                         `gTATAAACTTTCAGACGAAGACGTAATGGCAATCATGGAGTCTAGCGAGTCCAATATATCCTTAGCTCGCACCTATGG‑TGTCTCCCAACAGACTATTTGTGATATACGCAAAGGGAGGCGACATGGCAGGTTACGGCGCTAAAGGAATCCGAAAGGTTGGAGCGTTTCGCTCTGGCCTAGAGGACAAGGTTTCAAAGCAGTTGGAATCAAAAGGTATTAAATTCGAGTATGAAGAGTGGAAAGTGCCTTATGTAATTCAGGCGAGCAATCACACTTACACTCCAGACTTCTTACTTCCaaa                                                     >  1:379262/1‑301 (MQ=255)`  
                                                                                                                                                                                                                                                          `aataAACTTTCAGACGAAGACGTAAGGGCAAGCATGGAGTCCAGCGAGTCCAATGTATCCTTAGCTCGCACCTATGG‑TGTCTCCCAACAGACTATTTGTGAGATACGCAAAGGGAGGCGACATGGCAGGTTACGGCGCTAAAGGAATCCGAAAGGTTGGAGCGTTTCGCTCTGGCCTAGAGGACAAGGTTTCAAAGCAGTTGGAATCAAAAGGTATTAAATTCGAGTATGAAGAGTGGAAAGTGCCTTATGTAATTCCGGCGAGCAATCACACTTACACTCCAGACTTCTTACTTCCAAAc                                                    <  1:443242/300‑1 (MQ=255)`  
                                                                                                                                                                                                                                                                 `tttCAGACGAAGACGTAATGGCAATCAAGGAGTCCAGCGAGGCCAATGTATCCTTAGTTCGCAGCTATGG‑TGTCTCTAAACATACTATATGTGATATACGCAAAGGGAGGCGACATGGCAGGTTACGTCGCTAAAGGAATCCGAAAGGGTGAAGCGTTGCTCTCTGTCCAAGAGGACAAGGTTTCAAAGCAGTTGGAATC‑AAAGGTATTAAATTCGAGTATGAAGAGAGGAAAGTGCCTTATGTAATTCCGGCGAGCAATCACACTTACACTCCAGACTTCTTACTTCCAAACGGTATAtt                                            <  1:482952/301‑1 (MQ=255)`  
                                                                                                                                                                                                                                                                  `tacAGACGAAGACGTAATGGCAATCATGGAGTCCAGCGAGTCCAATGTATCCTTAGCTCGAACCTATGG‑TGTCTCACAACAGACTATTTGTGATATACGCAAAGGGAGGCGACATGGCAGGTTACGGCGCTAAAGGAATCCGAAAGGTTGGAGCGTTTCGCTCTGGCCTAGAGGAAAAGGTTTCAAAGCAGTTGGAATCAAAAGGTATTAAATTCGAGTATGAAGAGTGGAAAGTGCCTTATGTAATTCCGGCGAGCAATCACACTTACACTCCAGACTTCTTACTTCCAAACGGTATAtt                                            <  1:418826/299‑1 (MQ=255)`  
                                                                                                                                                                                                                                                                    `caaaCGAAGAATTGATGGCAATCATGGATTCCAGTGAGTCCAATGGATCCTTAGCTCGCACATATGG‑TGTCTCCCAACATATTATTTGTGATATACGCAAAGGGAGGCGACATGGCAGGTTACGGCGCTAAAGGAATCCGAAGGTTTGGAGGGTTTCGCTCTGGCCTAGAGGAAAAGGTTTCAAAGCAGTTGGAATCAAAAGGTATTAAATTCGAGTATGAAGAGTGGAAAGTGCCTTATGTAATTCCGGCGAGCAATCACACTTACACTCCAGACTTCTTACTTCCAAACGGTATATTct                                          <  1:16025/298‑2 (MQ=255)`  
                                                                                                                                                                                                                                                                    `cAGACGAAGACGTAATGGCAATCATGGAGTCCAGCGAGTCCAATGTATCCTTAGATCGCACCTATGG‑TGTCTCCCAACAGACTATTTGTGATATACGCAAAGGGAGGCGACATGGCAGGTTACGGCGCTAAAGGAATCCGAAAGGTTGGAGCGTTTCGCTCTGGCCTAGAGGACAAGGTTTCAAAGCAGTTGGAATCAAAAGGTATTAAATTCGAGTATGAAGAGTGGAAAGTGCCTTATGTAATTCCGGCGAGCAATCACACTTACACTCCAGACTTCTTACTTCCAAACGGTATATTCg                                          <  1:439533/301‑1 (MQ=255)`  
                                                                                                                                                                                                                                                                     `agagacaGACGTAAAGGAAATCAGTGAGTCCAGCGAGTCCAATGTATCCTTAGCTCGCACCTATGG‑TGTCTCCCAACAGACTATTTGTTATATACGCAAAGGGAGGCGACATGGCAGGTTACGGCGCTAAAGGAATCCGAAAGGTTGGAGCGTTTCGCTCTGGCCTAGAGGACAAGGTTTCAAAGCAGTTGGAATCAAAAGGTATTAAATTCGAGTATGAAGAGTGGAAAGTGCCTTATGTAATTCCGGCGAGCAATCACACTTACACTCCAGACTTCTTACTTCCAAACGGTATATTCGt                                         <  1:108102/295‑1 (MQ=255)`  
                                                                                                                                                                                                                                                                            `gACGTAATGGCAATCATGGAGTCCAGCGAGTCCAATGTATCCTTAGCTCGCACCTATGG‑TGTCTCCCAACAGACTATTTGTGATATACGCAAAGGGAGGCGACATGGCAGGTTACGGCGCTAAAGGAATCCGAAAGGTTGGAGCGTTTCGCTCTGGCCTAGAGGACAAGGTTTCAAAGCAGTTGGAATCAAAAGGTATTAAATTCGAGTATGAAGAGTGGAAAGTGCCTTATGTAATTCCGGCGAGCAATCACACTTACACTCCAGACTTCTTACTTCCAAACGGTCTATTCGTTGAGACa                                  >  1:257149/1‑301 (MQ=255)`  
                                                                                                                                                                                                                                                                            `gACGTAATGGCAATCATGGAGTCCAGCGAGTCCAATGTATCCTTAGCTCGCACCTATGG‑TGTCTCCCAACAGACTATTTGTGATATACGCAAAGGGAGGCGACATGGCAGGTTACGGCGCTAAAGGAATCCGAAAGGTTGGAGCGTTTCGCTCTGGACTAGAGGACAAGGTTTCAAAGCAGTTGGAATCAAAAGGTATTAAATTCGAGTATGAAGAGTGGAAAGTGCCTTATGTAATTCCGGCGAGCAATCACACTTACACTCCAGACTTCTTACTTCCAAACGGTATATTCGTTGTGACa                                  >  1:150940/1‑301 (MQ=255)`  
                                                                                                                                                                                                                                                                                    `ggCAATCATGGAGTCCAGCGAGTCCAATGTATCCTTAGCTCGCACCTATGG‑TGTCTCCCAACAGACTATTTGTGATATACGCAAAGGGAGGCGACATGGCAGGTTACGGCGCTAAAGGAATCCGAAAGGTTGGAGCGTTTCGCTCTGGCCTAGAGGACAAGGTTTCAAAGCAGTTGGAATCAAAAGGTATTAAATTCGAGTATGAAGAGTGGAAAGTGCCTTATGTAATTCCGGCGAGCAATCACACTTACACTCCAGACTTCTTACTTCCAAACGGTATATTCGTTGAGACAAAGGGTCt                          >  1:49116/1‑301 (MQ=255)`  
                                                                                                                                                                                                                                                                                           `aTGGAGTCCAGCGAGTCCAATGTATCCTTAGCTCGCACCTATGG‑TGTCTCCCAACAGACTATTTGTGATATACGCAAAGGGAGGCGACATGGCAGGTTACGGCGCTAAAGGAATCCGAAAGGTTGGAGCGTTTCGCTCTGGCCTAGAGGACAAGGTTTCAAAGCAGTTGGAATCAAAAGGTATTAAATTCGAGTATGAAGAGTGGAAAGTGCCTTATGTAATTCCGGCGAGCAATCACACTTACACTCCAGACTTCTTACTTCCAAACGGTATATTCGTTGAGACAAAGGGTCTGTGGGaa                   >  1:502892/1‑301 (MQ=255)`  
                                                                                                                                                                                                                                                                                               `aGTCCAGCGAGTCCAATGTATCCTTAGCTCGCACCTATGG‑TGTCTCCCAACAGACTATTTGTGATATACGCAAAGGGAGGCGACATGGCAGGTTACGGCGCTAAAGGAATCCGAAAGGTTGGAGCGTTTCGCTCTGGCCTGGAGGACAAGGTTTCAAAGCAGTTGGAATCAAAAGGTATTAAATTCGAGTATGAAGAGTGGAAAGTGCCTTATGTAATTCCGGCGAGCAATCACACTTACACTCCAGACTTCTTACTTCCAAACGGTATATTCGTTGAGACAAAGGGTCTGTGGGAAAGCg               >  1:272831/1‑301 (MQ=255)`  
                                                                                                                                                                                                                                                                                                    `aGCGAGTCCAATGGATCCTTAGCTCGCACCTATGG‑TGTCTCCCAACAGACTATTTGTGATATACGAAAAGGGAGGCGACATGGCAGGTTACGGCGCTAAAGGAATCCGAAAGGTTGGAGCGTTTCGCTCTGGCCTAGAGGACAAGGTTTCAAAGCAGTTGGAAGCAAAAGGTATTAAATTCGAGTATGGAGAGTGGAAAGTGCCTTATGTAATTCCGGCGAGCAATCACACTTACACTCCAGACTTCTTACTTCCAAACGGTATATTCGTTGAGACAAAGGGTCTGTGGGAAAGCgatgat          <  1:239834/301‑1 (MQ=255)`  
                                                                                                                                                                                                                                                                                                        `aGTCCAATGTATCCTTAGCTCGCACCTATGG‑TGTCTCCCAACAGACTATTTGTGATATACGCAAAGGGAGGCGACATGGCAGGTTACGGCGCTAAAGGAATCCGAAAGGTTGGAGCGTTTCGCTCTGGCCTAGAGGACAAGGTTTCAAAGCAGTTGGAATCAAAAGGTATTAAATTCGAGTATGAAGAGTGGAAAGTGCCTTATGTAATTCCGGCGAGCAATCACACTTACACTCCAGACTTCTTACTTCCAAACGGTATATTCGTTGAGACAAAGGGTCTGTGGGAAAGCgactgtctct      >  1:262100/1‑293 (MQ=255)`  
                                                                                                                                                                                                                                                                                                            `cAATGTATCCTTAGCTCGCACCTATGG‑TGTCTCCCAACAGACTATTTGTGATATACGAAAAGGGAGGCGACATGGCAGGTTACGGCGCTAAAGGAATCCGAAAGGTTGGAGCGTTTCGCTCTGGCCTAGAGGACAAGGTTTCAAAGCAGTTGGAATCAAAAGGTATTAAATTCGAGTATGAAGAGTGGAAAGTGCCTTATGTAATTCCGGCGAGCAATCACACTTACACTCCAGACTTCTTACTTCCAAACGGTATATTCGTTGAGACAAAGGGTCTGTGGGAAAGCGATGATagaaagaa  >  1:507705/1‑301 (MQ=255)`  

                                                                                                                                                                                                                                                                                                            `|`                                                                                                                                                                                                                                                                                                               
`AATCAAGGTGACTTCCTCTGGGTGTTGGGAGTGGCAGGGCGCTACGAACAATAAAGGGTACGGGCAGGTGTGGTGCAGCAATACCGGAAAGGTTGTCTACTGTCATCGCGTAATGTCTAATGCTCCGAAAGGTTCTACCGTCCTGCACTCCTGTGATAATCCATTATGTTGTAACCCTGAACACCTATCCATAGGAACTCCAAAAGAGAACTCCACTGACATGGTAAATAAGGGTCGCTCACACAAGGGGTATAAACTTTCAGACGAAGACGTAATGGCAATCATGGAGTCCAGCGAGTCCAATGTATCCTTAGCTCGCACCTATGG‑TGTCTCCCAACAGACTATTTGTGATATACGCAAAGGGAGGCGACATGGCAGGTTACGGCGCTAAAGGAATCCGAAAGGTTGGAGCGTTTCGCTCTGGCCTAGAGGACAAGGTTTCAAAGCAGTTGGAATCAAAAGGTATTAAATTCGAGTATGAAGAGTGGAAAGTGCCTTATGTAATTCCGGCGAGCAATCACACTTACACTCCAGACTTCTTACTTCCAAACGGTATATTCGTTGAGACAAAGGGTCTGTGGGAAAGCGATGATAGAAAGAA  >  NC_001604.1/9886‑10486`

  

Alignment Legend

Aligned base mismatch/match (shaded by quality score): `ATCG/ATCG` `< 3 ≤` `ATCG/ATCG` `< 10 ≤` `ATCG/ATCG` `< 22 ≤` `ATCG/ATCG` `< 34 ≤` `ATCG/ATCG` `< 38 ≤` `ATCG/ATCG`

Unaligned base: `atcg`    Masked matching base: `atcg`    Alignment gap: `‑`    Deleted base: `‑`

Reads not counted as support for junction

read\_name Not counted due to insufficient overlap past the breakpoint.

read\_name Not counted due to not crossing MOB target site duplication.
